# Prevalence of Urinary Incontinence in Female Residents of American Nursing Homes and Association with Neuropsychiatric Disorders

**DOI:** 10.4021/jocmr2009.04.1232

**Published:** 2009-04-14

**Authors:** Mohammad Sami Walid

**Affiliations:** Medical Center of Central Georgia, 840 Pine Street, Suite 950, Macon, GA 31201, USA. Email: mswalid@yahoo.com

## Abstract

**Background:**

Urinary incontinence (UI) is most common in older women.

**Methods:**

We studied the prevalence of UI among female residents of nursing homes and the influence of associated neuropsychiatric problems on the rates of UI using the results of the 2004 National Nursing Home Survey (NNHS).

**Results:**

Analysis shows that 37% of female nursing home residents are incontinent, especially those with dementia. Residents with depression or schizophrenia are also more likely to have UI whereas those with anxiety, paranoia, or obsessive-compulsive disorder have less UI rates. There are significant associations with neuropsychiatric disorders except for bipolar disease.

**Conclusions:**

We recommend prioritizing behavioral interventions and environmental manipulations for female residents with dementia, depression, and schizophrenia to increase the cost-effectiveness of UI management programs in nursing homes.

**Keywords:**

Urinary incontinence; Female residents; Nursing home; Neurodegenerative; Psychiatric

## Introduction

Urinary incontinence (UI) is most common in older women. The prevalence of UI in women living in the community increases with age, up to 29% at age 80 years or older [1]. In nursing homes, the prevalence of UI is expected to be higher than in the community due to impaired mobility and other factors. In this paper, we studied the prevalence of UI among female residents of American nursing homes and the influence of associated neuropsychiatric problems on the rates of UI.

## Materials and Methods

We used the results of the 2004 National Nursing Home Survey (NNHS) conducted by the National Center for Health Statistics (NCHS) and provided by the Centers for Disease Control and Prevention (CDC) on their website [2]. The survey includes data on 1317292 residents, over 65 years, and 241 variables. We recoded the bladder control variable to continent (continent, usually continent, occasionally incontinent), incontinent (frequently incontinent, incontinent) and missing (do not know, not ascertained) and studied its relationship with the following neurodegenerative and psychiatric entities:

- Dementia (ICD-9 code 290, 331)
- Schizophrenia (ICD-9 code 295 297 298),
- Paranoia (ICD-9 code 297),
- Depression (ICD-9 code 311 296.2 296.3 300.4),
- Bipolar disease (ICD-9 code 296),
- Anxiety (ICD-9 code 300 300.1 300.2 300.5 300.8 300.9),
- Obsessive-compulsive disorder OCD (ICD-9 code 300.3).

Data were analyzed using the Pearson Chi-Square Test of Independence with the help of the SPSS v16 software.

## Results

Analysis of valid data shows that 37% of female nursing home residents are incontinent. Patients with dementia (n=283904), depression (n=459269), and schizophrenia (n=146462) are more likely to have UI whereas those with anxiety (n=153573), paranoia (n=27137), or obsessive-compulsive disorder (n=5063) have less UI rates (Table 1). There are significant associations with neuropsychiatric problems except for bipolar disease (n=24206) as shown in Table 2. Bipolar disease does not make a difference for UI rate (p>0.05). Dementia has the highest chi-square statistic among neuropsychiatric factors favoring UI followed by depression and schizophrenia whereas anxiety has the highest chi-square statistic among factors hindering UI followed by paranoia and obsessive-compulsive disorder.

**Table 1:** Rates of UI associated with neurodegenerative and psychiatric disorders.

	Dementia		Schizophrenia		Paranoia		Depression		Bipolar Disease		Anxiety		OCD	
	Yes	No	Yes	No	Yes	No	Yes	No	Yes	No	Yes	No	Yes	No
UI (%)	48.5	33.3	38.0	36.6	31.2	36.8	37.5	36.3	36.7	36.7	31.2	37.6	31.7	36.7

**Table 2:** Associations between UI and neurodegenerative and psychiatric disorders

		Dementia	Schizophrenia	Paranoia	Depression	Bipolar Disease	Anxiety	OCD
UI	Χ^2^	17076.929	85.853	276.658	143.407	.002	1940.566	44.434
	df	1	1	1	1	1	1	1
	Sig.	.000*	.000*	.000*	.000*	.968	.000*	.000*

The Chi-square statistic is significant at the 0.05 level.

## Discussion

UI is a difficult geriatric problem and mainly a problem of elderly women [3, 4]. The pathogenesis of urinary incontinence in older female patients is multifactorial. Age-related physiologic changes, pelvic floor prolapse, neurological and psychiatric disorders frequently contribute to the etiology of incontinence altogether.

The U.S. population is projected to increase from 285 millions in 2000 to 335 millions by 2020 with more than 20% of the population over 60. With the aging population there is going to be greater demand for nursing homes and higher rates of UI. Ideally, treating UI in the community is preferred, which helps prevent or delay institutionalization and offset some of the expenses off the healthcare system. UI often have negative effects on the lives of elderly women and their relatives who suffer physical discomfort, embarrassment, stigma, social isolation, and the financial costs of treatment. UI can, thus, be an additional reason for admission to a nursing home [5]. On the other hand; newly admitted residents are at risk of developing problems with continence de novo [6].

The prevalence of UI in female residents of nursing homes is, indeed, higher than in the community as the above results show. UI usually becomes a concern for nursing home staff whose job includes helping residents prevent or curb their incontinence. In some cases, incontinence problems may be corrected simply by treating the underlying problem. This approach generally works for incontinence caused by hyperglycemia or excess fluid intake. With neurodegenerative and psychiatric diseases the mechanism of UI may be different. According to the above results, paranoid and neurotic patients are in better control of their bladder than patients with neurodegenerative and schizoaffective disorders except for bipolar patients who do not show any difference in UI rate from patients without bipolar disease. We, thus, recommend prioritizing behavioral interventions and environmental manipulations for female patients with dementia, depression, and schizophrenia. This includes regular voiding, regular reviewing of prescribed medicines to reduce polyuric side effects, and removal of architectural barriers. This will probably increase the cost-effectiveness of UI management programs in nursing homes.

## References

[1] (2007). NIH state-of-the-science conference statement on prevention of fecal and urinary incontinence in adults. NIH Consens State Sci Statements.

[2] National Nursing Home Survey. National Center for Health Statistics. Accessed 11/14/2008. URL: http://www.cdc.gov/nchs/nnhs.htm.

[3] Beck RP, Kase NG, Weingold AB (1983). Pelvic relaxation prolapse. Principles and practice of clinical gynaecology.

[4] Olsen AL, Smith VJ, Bergstrom JO, Colling JC, Clark AL (1997). Epidemiology of surgically managed pelvic organ prolapse and urinary incontinence. Obstet Gynecol.

[5] Morrison A, Levy R (2006). Fraction of nursing home admissions attributable to urinary incontinence. Value Health.

[6] Boguth K, Schenk L (2008). [New-onset urinary incontinence in the first six month after admission into a nursing home: prevalence, incidence and remission, risk and protective factors]. Z Gerontol Geriatr.

